# Analysis and prediction of the axial compression properties of desert sand concrete with steel tube restraint based on an improved BP neural network model

**DOI:** 10.1371/journal.pone.0332585

**Published:** 2025-09-22

**Authors:** Wang Xiaoqian, Li Xinyang, Chen Yuzhi, Qiu Xiangyu, Song Ling

**Affiliations:** 1 School of Architectural Engineering, Jinling Institute of Technology, Nanjing, Jiangsu, China; 2 School of Civil Engineering, Anhui Jianzhu University, Hefei, Anhui, China; 3 School of Mechanics and Materials, Hohai University, Nanjing, Jiangsu, China; 4 School of Architecture and Engineering, Nantong Vocational University, Nantong, Jiangsu, China; Graphic Era Deemed to be University, INDIA

## Abstract

Accurate analysis and prediction of axial compression are important for ensuring the construction quality and safety of desert sand recycled aggregate concrete confined by steel tubes. In this study, the axial compressive strength and elastic modulus of recycled aggregate concrete with different sand contents, water–cement ratios, and steel constraints were tested to evaluate the effects of these factors on the axial compressive performance of the recycled aggregate concrete. It was determined that a steel tube restraint could effectively improve the ductility of desert sand recycled aggregate concrete. However, with increases in the sand content and water–cement ratio, the peak stress slightly decreased. The axial compressive strength and elastic modulus of the recycled sand aggregate concrete confined by steel tubes exhibited little change in the elastic stage under a functional load. During the initial stage of loading, the lateral strain exhibited strong discrete characteristics. In the peak stress stage, the transverse coefficient gradually increased. Overall, our analysis revealed that axial compressive performance exhibits evident engineering uncertainty under the comprehensive influence of factors such as steel constraint, desert sand content, and water–cement ratio. Therefore, an improved backpropagation (BP) neural network model of the axial compressive properties of recycled aggregate concrete with steel-tube-confined sand was established with the presence of steel constraints, desert sand content, and water–cement ratio serving as inputs, and axial compression strength and elastic modulus as outputs. Engineering verification calculations indicated that the BP neural network model can predict concrete performance under actual working conditions with a small error rate. Compared with traditional models, the neural network model has comprehensive advantages in terms of fitting accuracy, reduced overfitting, and enhanced stability.

## 1. Introduction

In recent years, with the accelerated development of infrastructure construction, concrete production demand has increased and the required resources such as sand and gravel have become increasingly scarce. River sand, which is the most widely used fine aggregate in concrete mixtures, is indispensable in the construction of engineering facilities. Desert sand has been considered to replace or partially replace river sand in the preparation of desert sand concrete to alleviate concerns regarding desertification and the mismatch between the supply and demand of sand in engineering construction. China has a large desert area with a wide distribution. Therefore, utilising desert sand concrete can not only meet the development goal of low-carbon concrete but also circumvent the dwindling supply of river sand. Recycled aggregate concrete can reduce the environmental pollution associated with waste concrete and save considerable resources, in line with the concepts of sustainable development. However, owing to the inevitability of cracks and warping in old mortar used in the production process of recycled aggregate, it has the disadvantages of a high crushing value and high water absorption. As a result, the strength of concrete prepared with recycled aggregates is often reduced. In engineering practice, it has been found that the particle size of desert sand exhibits significant uncertainty compared with natural river sand, leading to engineering uncertainties in the workability and mechanical properties of prepared desert recycled aggregate concrete [1,2].

The mixture design and mechanical properties of desert sand recycled aggregate concrete have been researched extensively [3–5]. Mohammad et al. [6] aimed to develop four types of sustainable modified sand mixtures by using different proportions of desert sand, natural crushed sand, and recycled crushed sand. While evaluating the physical and chemical properties of the modified sand, four concrete mixture ratios were also prepared using the newly developed modified sand. Twelve groups of hybrid fibre-reinforced desert sand concrete column specimens were tested by Hou et al. [7] and the effects of the fibre parameters on the failure mode, stress–strain curve, compressive strength, peak strain, elastic modulus, and toughness were studied. Qu et al. [8] used scanning electron microscopy, acoustic emission detection, and other technologies to analyse the microstructures of various samples and identify damage processes. They found that desert sand yielded a refined microstructure, filled pores, and good comprehensive properties when the substitution rate was 40%.

In steel-tube-confined concrete, steel tubes are used to restrain the lateral deformation of core concrete structures [9,10]. The steel tube does not directly bear the longitudinal load but passively bears force through the bonding friction between its inner wall and the concrete, thereby providing a restraining function for the core concrete. Following the compression of concrete-filled steel tube short columns, the core concrete is in a three-dimensional compressive stress state, and the compressive strength and ultimate compressive strain significantly improve [11–13]. Significant research has been conducted on concrete-filled steel tubes and recycled concrete-filled steel tubes. Based on the shear friction mechanics of partial interaction, Hao et al. [14] established a passive stress–strain model for short steel-tube-constrained concrete columns. This model predicts the ultimate strength of concrete-constrained steel tubes and simplifies analysis into a design-oriented approach. Additionally, a parametric study was conducted to explore the influence of specimen size on the performance of steel-tube-constrained concrete columns. Chen et al. [15] conducted axial compression tests on 12 short thin-walled steel tubes to study the effects of the length-to-slenderness ratio, width-to-thickness ratio of reinforced and unreinforced thin-walled steel tubes, and thickness of reinforced high-ductility concrete on the performance of thin-walled steel tubes. The ultimate bearing capacity, failure mode, and local buckling behaviour of steel tubes were evaluated. However, there has been little research on steel-tube-confined concrete using desert sand recycled aggregate. Additionally, previous research on recycled concrete has not fully considered the uncertainty distribution of the pressure properties of steel-tube-confined concrete in actual engineering applications [16–19], which may lead to errors and accidents.

In this study, axial compression tests of steel-tube-confined desert sand recycled aggregate concrete (STCDSRAC) specimens were conducted and the influence of different desert sand contents on the mechanical properties of STCDSRAC was analysed while considering engineering uncertainty. Based on the results, an improved backpropagation (BP) neural network model was trained to predict concrete performance, providing an effective reference for the engineering application of STCDSRAC.

## 2. Testing overview

### 2.1. Testing materials and parameters

The cement used in our axial compression tests was PO 42.5 ordinary Portland cement of the Conch brand, and the ore powders were S95 ground and granulated blast-furnace slag powder. The specific surface areas of the cement and mineral powder were 378 and 420 m^2^/kg, respectively. The chemical compositions of the cement and mineral powders are listed in Table 1.

**Table 1:** Chemical compositions of cementitious materials (%).

Component	CaO	MgO	SiO_2_	Fe_2_O_3_	P_2_O_5_	Al_2_O_3_	SO_3_	Loss on ignition
Mineral powder	45.09	6.99	27.33	0.45	0.13	13.66	4.03	0.95
Cement	54.65	2.58	22.07	4.32	1.03	6.30	2.59	2.14

**Fig 1:**
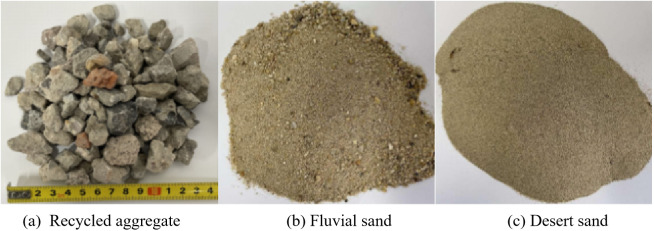
Coarse and fine aggregates used for testing.

**Fig 2:**
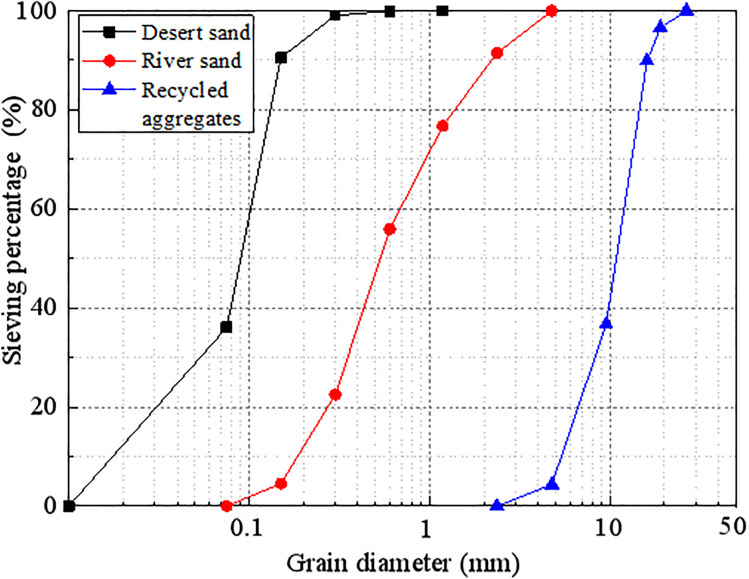
Aggregate characteristic curves.

The coarse aggregate was recycled aggregate with a maximum nominal particle size of 26.5 mm, loose bulk density of 1264.5 kg/m^3^, water absorption rate of 4.9%, and crushing index of 15.2%. The obtained results reached a Class II crushing index. To alleviate the resource shortage problems associated with using river sand as a fine aggregate in concrete, the fine aggregates consisted of a mixture of natural river sand and desert sand from the Taklimakan Desert. The fineness modulus of the natural river sand was 2.486 and that of the desert sand was 0.106. The coarse and fine aggregates are presented in Fig 1 and the aggregate grading curve is presented in Fig 2.

**Fig 3:**
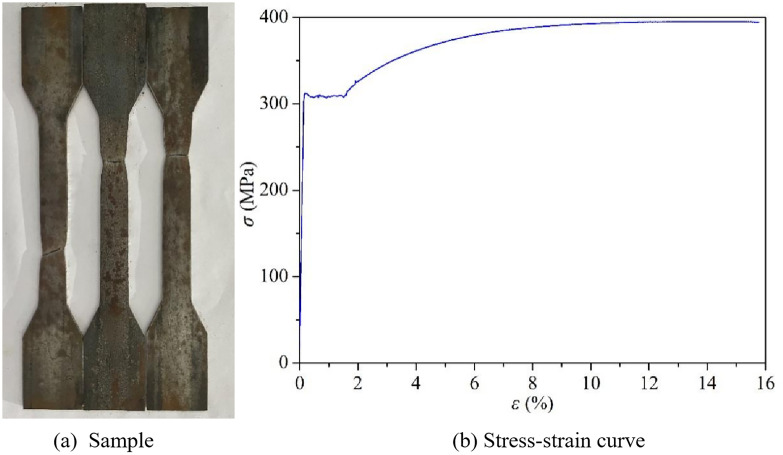
Steel tube material property test.

Steel pipe dimensions and material properties: The inner diameter of the steel pipe is 150 mm, the wall thickness is 3 mm, and the height is 290 mm. By shearing and processing the steel pipe into “dumbbell-shaped” specimens and conducting uniaxial tensile tests on the steel, the tensile stress-strain curves were obtained, as shown in Fig 3. The measured yield strength of the steel pipe is 306.6MPa, and the elastic modulus is 196.5GPa.

### 2.2. Design of concrete specimen mixture proportions and steel tube restraint

Four groups of desert sand recycled aggregate concrete specimens and 12 groups of STCDSRAC specimens were prepared, resulting in a total of 16 groups of cylindrical specimens with three specimens in each group. The sand ratio of the concrete specimens was 0.46, rate of cement replacement with the mineral powder admixture was 50%, and water-reducing agent content was equal to 0.7% of the cementitious material. The test piece numbering rules were based on whether the test pieces had steel tube constraints, the proportion of desert sand replacing river sand, and water–cement ratio of the concrete. Considering DSRACD0(W30) and STCDSRACD60(W40) as examples, DSRACD0(W30) indicates a specimen without steel tube constraint or desert sand and with a water–cement ratio of 30%. STCDSRACD60(W40) indicates a specimen with steel tube constraint, 60% desert sand, and a 40% water–cement ratio. The specific test piece groups and their properties are listed in Table 2.

**Table 2:** Mixture proportions and properties of the concrete specimens.

Specimen number	Cement (kg/m³)	Slag (kg/m³)	River sand (kg/m³)	Desert sand (kg/m³)	Recycled aggregate (kg/m³)	Water (kg/m)	Water reducer (kg/m³)	Steel-constraint thickness (mm)
DSRACD0(W30)	241.5	241.5	815	0	957	145	3.42	0
DSRACD20(W30)	241.5	241.5	652	163	957	145	3.42	0
DSRACD40(W30)	241.5	241.5	489	326	957	145	3.42	0
DSRACD60(W30)	241.5	241.5	326	489	957	145	3.42	0
STCDSRACD0(W30)	241.5	241.5	815	0	957	145	3.42	3
STCDSRACD20(W30)	241.5	241.5	652	163	957	145	3.42	3
STCDSRACD40(W30)	241.5	241.5	489	326	957	145	3.42	3
STCDSRACD60(W30)	241.5	241.5	326	489	957	145	3.42	3
STCDSRACD0(W35)	241.5	241.5	815	0	957	145	3.42	3
STCDSRACD20(W30)	241.5	241.5	652	163	957	145	3.42	3
STCDSRACD40(W30)	241.5	241.5	489	326	957	145	3.42	3
STCDSRACD60(W30)	241.5	241.5	326	489	957	145	3.42	3
STCDSRACD0(W40)	241.5	241.5	815	0	957	145	3.42	3
STCDSRACD20(W40)	241.5	241.5	652	163	957	145	3.42	3
STCDSRACD40(W40)	241.5	241.5	489	326	957	145	3.42	3
STCDSRACD60(W40)	241.5	241.5	326	489	957	145	3.42	3

**Fig 4:**
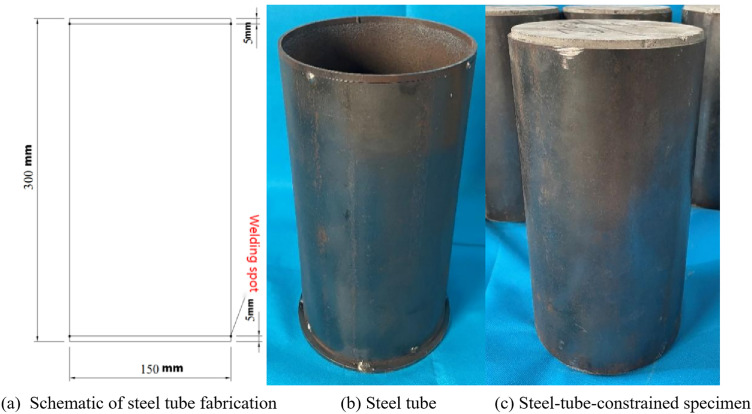
Preparation process of steel-tube-constrained concrete specimens.

To prepare the specimens, steel tube with a wall thickness of 3 mm was cut to a length of 290 mm. Additional rings with a length of 5 mm were spot-welded onto both ends and one end of the steel tube was sealed using a spot-welded steel plate. After the concrete was poured, the welds were ground off, and the rings and bottom plate were removed to obtain steel-tube-constrained concrete specimens, as shown in Fig 4. After each specimen was poured, it was transferred to a curing room and cured for 28 d under standard conditions [20,21].

**Fig 5:**
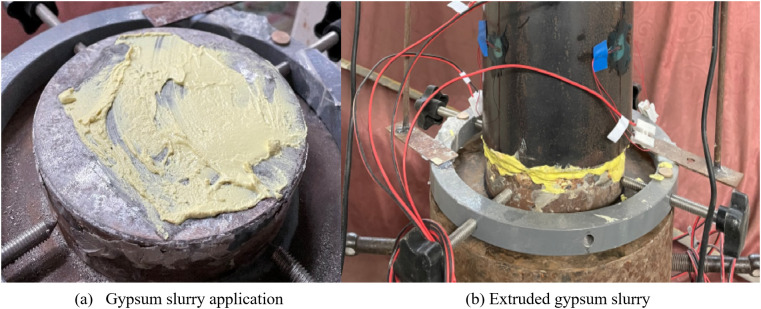
Loading setup for cured samples.

Axial compression testing required precisely flat loading surfaces on the specimens. An uneven loading surface would induce local contact between the specimen and loading plate, causing the specimen to bear a split tension function and significantly reducing the ultimate load [22–24]. Therefore, prior to testing, the end surfaces of each specimen were ground with a grinder and a high-strength gypsum powder and water slurry was applied as a coating between each specimen and the loading plate. The excess gypsum slurry was extruded by applying a preload of approximately 10 kN before the gypsum hardened to ensure dense contact between the specimen and the loading plate, as shown in Fig 5.

### 2.3. Layout of measurement points and loading scheme

**Fig 6:**
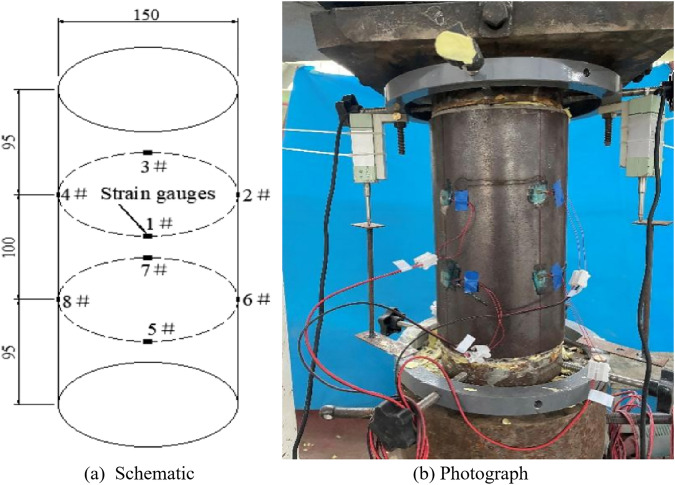
Measurement point arrangement and loading device.

**Fig 7:**
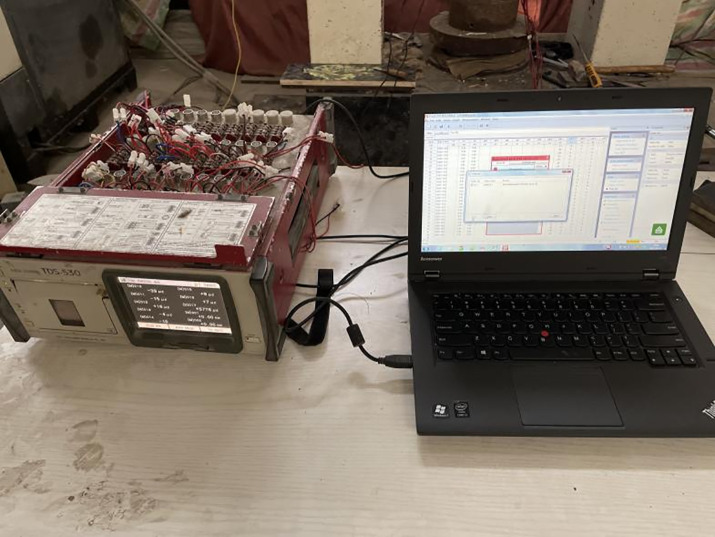
TDS530 Static strain gauge.

**Fig 8:**
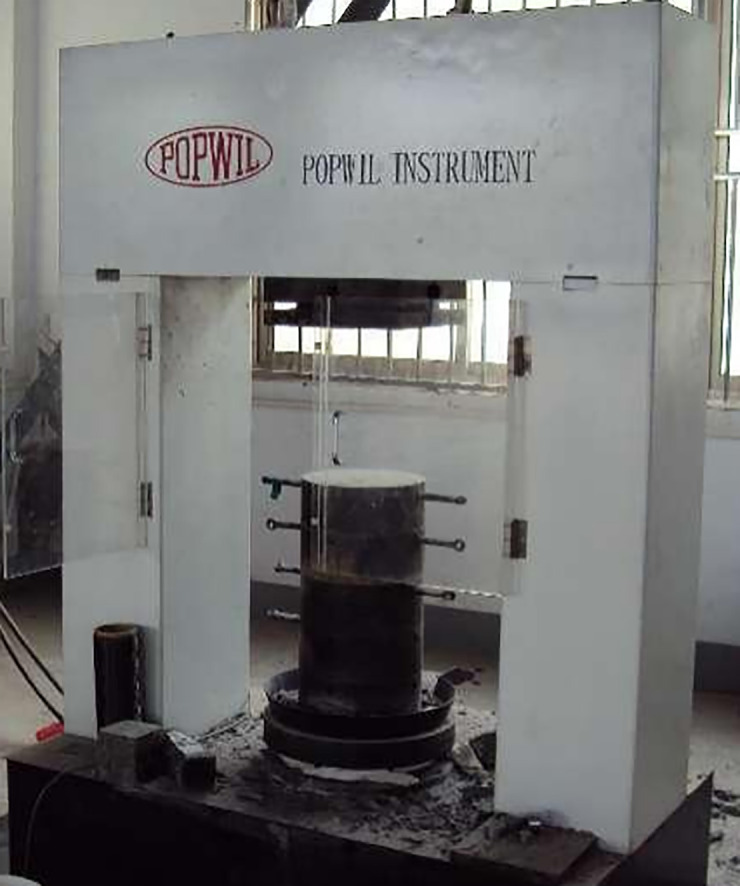
High-stiffness hydraulic servo pressure-testing machine.

As shown in Fig 6, four circumferential strain gauges were attached at the upper and lower one-third heights of the steel tube to measure its lateral deformation under an axial compression load. Additionally, two linear variable differential transformer (LVDT) displacement sensors were installed on the steel plates loaded on both ends of the specimen to measure the axial deformation of the specimen. Data acquisition was performed using a TDS530 static strain gauge, as shown in Fig 7. Modern A/D converter technology ensured the accuracy and stability of data acquisition. During testing, the strain gauges and LVDTs were connected to the TDS530 static strain gauge, allowing the synchronous recording of loading and deformation data. In addition to the deformation of the core concrete, the data measured by the displacement meter included the deformation of the steel base plate at the loading end, which was subtracted from the calculation. The loading device was a high-stiffness hydraulic servo pressure-testing machine produced by a company in Hangzhou, with a maximum load of 3000 kN, as shown in Fig 8. The loading process utilised displacement control with a loading speed of 1 mm/min.

## 3. Test results and analysis

### 3.1. Stress–strain curves

**Fig 9:**
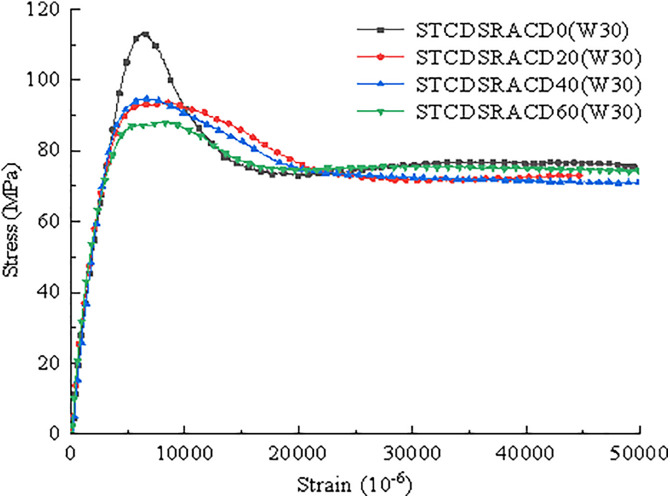
STCDSRAC axial compressive stress–strain curves.

According to the data processing method outlined in GB-T 5008 (2019 Standard for Test Methods of Mechanical Properties of Ordinary Concrete), when the difference between the maximum or minimum value and the intermediate value of one of the three measured values exceeds 15% of the intermediate value, the maximum and minimum values should be removed and the intermediate value should be considered as the strength value of a group of specimens. The axial compressive stress–strain curves of the STCDSRAC with different desert sand contents are presented in Fig 9. One can see that there are three stages in the axial compression stress–strain curves with a pronounced uncertainty distribution. The first stage is the elastic stage, where the axial deformation changes linearly with an increase in load and the stiffness is large. The second stage is the elastic–plastic stage, where the stiffness gradually decreases with an increase in load. The third stage is the plastic stage, where the stiffness of the members tends to be constant and the displacement growth rate increases. Compared with the control STCDSRACD0 specimen without desert sand, the peak stress of the STCDSRAC specimens with desert sand was reduced, and the strength degradation trend slowed after the peak stress was reached.

**Fig 10:**
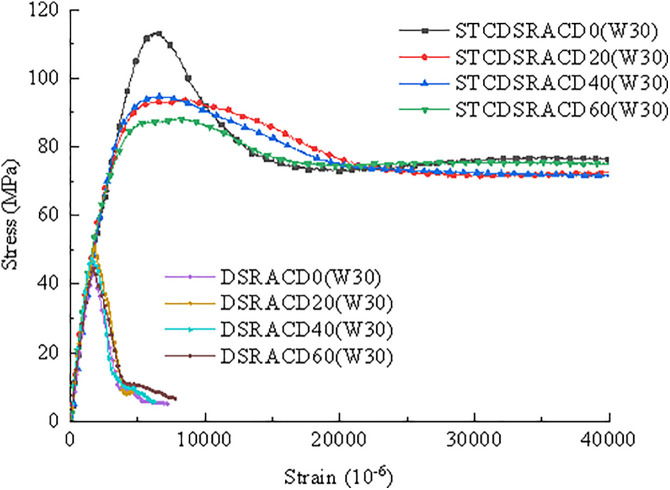
Comparison of stress–strain curves between STCDSRAC and DSRAC.

In Fig 10, the stress–strain curves of the STCDSRAC specimens exhibit no obvious descending segments, unlike the DSRAC specimens. The compressive strength of the STCDSRAC was reduced by the addition of desert sand. With an increase in desert sand content from 20% to 60%, the peak stress of STCDSRAC decreased by 6.7%.

**Fig 11:**
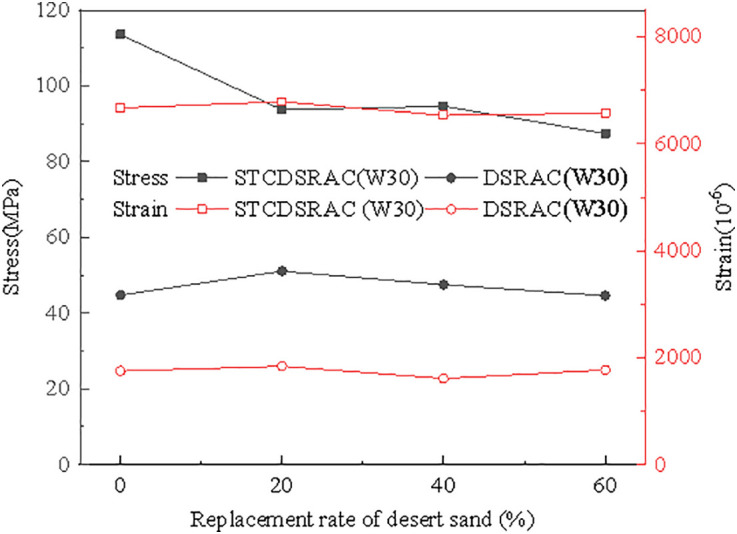
Peak strain and stress curves.

Fig 11 presents a graph of the peak stress and strain values, revealing that for STCDSRAC, the peak strain increases as the peak stress increases. The peak strains of STCDSRAC with 0%, 20%, 40%, and 60% desert sand increased by 280.7%, 269.0%, 305.4%, and 271.0%, respectively, compared with those of DSRAC specimens with the same desert sand content. Therefore, it can be concluded that steel tube restraint can significantly improve the ductility of desert sand recycled aggregate concrete, enhancing its deformation performance, which is accompanied by a clear engineering uncertainty distribution.

### 3.2. Uncertainty analysis

According to the results of preliminary tests, different axial compressive strengths were obtained. The axial compressive strengths of STCDSRAC and DSRAC specimens with different desert sand contents are listed in Table 3.

**Table 3:** Peak strain and stress values of the specimens.

Test piece No.	Peak stress (MPa)	Peak strain (με)	Test piece No.	Peak stress (MPa)	Peak strain (με)
STCDSRACD0(W30)	113.54	6677	DSRACD0(W30)	44.79	1754
STCDSRACD20(W30)	93.70	6790	DSRACD20(W30)	51.08	1840
STCDSRACD40(W30)	94.64	6535	DSRACD40(W30)	47.53	1612
STCDSRACD60(W30)	87.42	6577	DSRACD60(W30)	44.61	1773

One can see that compared with DSRAC specimens with the same desert sand content, the peak stress of the STCDSRAC specimens was significantly increased because the core concrete confined by steel tubes was in a three-dimensional compression state. The peak stress values of the STCDSRAC specimens with 0% to 60% desert sand increased by 153.5%, 83.4%, 99.1%, and 96.0%, in order, compared with the DSRAC specimens with the same desert sand contents. Overall, the results indicate that steel tube restraint can significantly increase the compressive strength of desert sand recycled aggregate concrete. However, the degree of improvement is not perfectly linear, exhibiting discrete characteristics.

To analyse the uncertainty distribution of the axial compression performance further, a reciprocating loading experiment was conducted on the STCDSRAC specimens. The loading scheme was as follows. Prior to reaching the peak load, force control loading was performed with four loading and unloading cycles. Each load was 20% of P_0_ (P_0_ represents the estimated peak load). After the peak load, displacement control loading was performed with three loading and unloading cycles at strains of 0.01, 0.02, and 0.03, in order.

**Fig 12:**
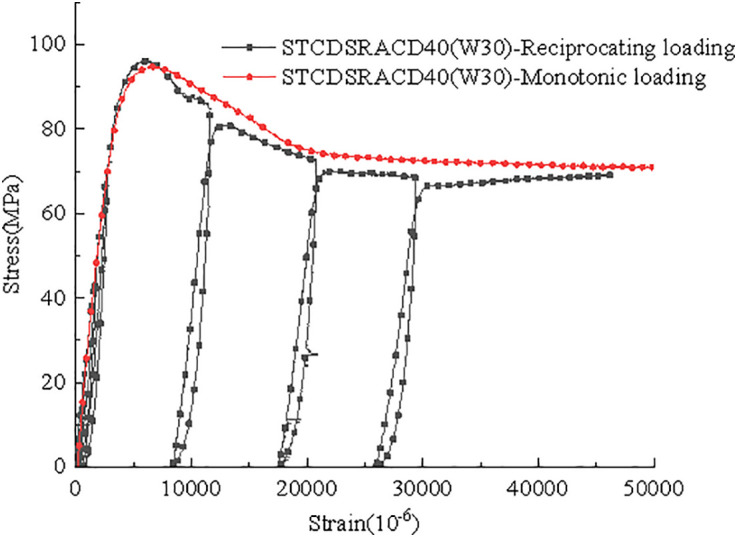
Stress–strain curves under reciprocating and monotonic loading.

Considering the STCDSRACD40(W40) specimen as an example, a comparison of the stress–strain curves under reciprocating and monotonic loading is presented in Fig 12. The trend lines of the reciprocating load specimen largely coincide with the curve of the monotonic load specimen, indicating that reciprocating loading does not lead to significant strength degradation.

The elastic modulus of the concrete was considered as the secant modulus at 0.4fu (peak stress) in the rising section of the stress–strain curve. The elastic moduli of STCDSRACD40(W40) under reciprocating loading are presented in Table 4. In general, the elastic modulus of the specimen under reciprocating and monotonic loading exhibited little change in the elastic stage. As the specimen entered the elastic–plastic stage, its unloading stiffness under reciprocating loading decreased gradually.

**Table 4:** Elastic moduli under reciprocating loading (taking STCDSRACD40(W30) as an example).

Loading phase	Elastic modulus (GPa)
Monotonic loading	34.3
0% → 40% P_0_	30.5
0% → 60% P_0_	31.5
0% → 80% P_0_	33.2
0.01	26.1
0.02	24.0
0.03	21.9

**Fig 13:**
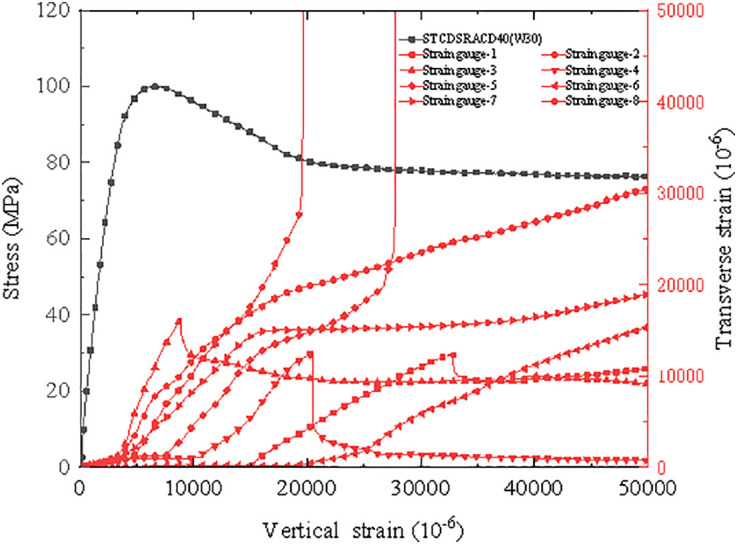
Transverse strain curves of the steel tube and stress–strain curve of the specimen.

According to the transverse strain data of the steel tube recorded by the strain gauge, the transverse strain of the steel tube and the stress–strain curve of the specimen were plotted. As shown in Fig 13, the circumferential strain gauge data for each part of the specimen are consistent in the elastic stage. Because the steel tube imposes a uniform lateral constraint function on the concrete core, there is a clear correspondence between the peak bearing capacity of the specimen and yield point of the steel tube. Additionally, in the initial stage of loading, the lateral strain was found to exhibit significant discreteness, which can be attributed to the small deformation of the specimen in the initial stage of loading and significant influence of engineering errors on the results. However, in the stage close to the peak stress, owing to the accumulation of numerous micro-cracks in the specimen, the growth of the lateral deformation accelerated, increasing the lateral coefficient.

## 4. BP neural network and improvement strategies

Considering the uncertain distribution of the axial compressive properties of recycled sand aggregate concrete confined by steel tubes, to obtain accurate and reliable axial compression and elastic modulus values to guide engineering practice, it is clearly inadequate to rely on experimental data fitting. Therefore, we implemented an improved artificial intelligence algorithm model to predict the compressive strength and splitting tensile strength of steel-tube-constrained concrete for the first time [25–28].

### 4.1. Traditional BP neural network

**Fig 14:**
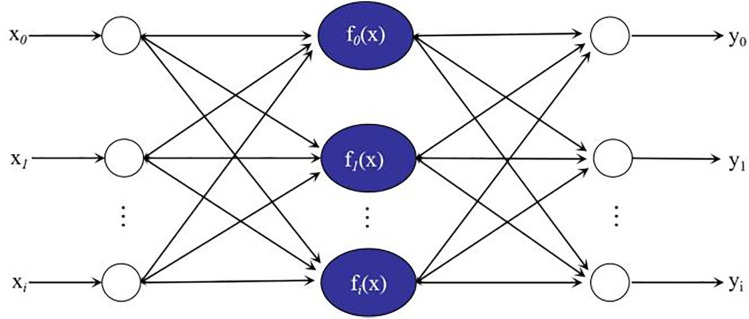
BP neural network architecture.

In 1986, Rumhardt proposed an intelligent error BP algorithm, which solved the problem of learning the layer connection weights of the hidden units in a multilayer neural network through the forward flow of information elements and feedback transmission of error elements, and established the BP neural network model [29]. The network topology consists of three types of layers, namely input, hidden, and output layers, as shown in Fig 14. The information flow of the model is divided into positive and negative feedback. In the initial calculation, the information flows in the traditional order of input layer → hidden layer → output layer. If there is an error between the output value and expected value, then negative feedback propagation is initiated, the error signal is returned along the original neurons, and the weights of the neurons in each layer are modified. This iteration is repeated until the error disappears or is within the allowable range.

The processing steps of the traditional BP neural network are as follows.

(1) The connection weights are initialised.

$$\omega_{sp}=Random(\cdot )$$ (1)

Here, *s* and *p* represent different nodes of the network neurons, ω represents the connection weight of the corresponding node, and *Random()* is a random value generator.

(2) According to the network model, *P* samples are inputted for network training and the input sequence number of the current sample is assumed to be *p*.
(3) The signal output of each layer is calculated using the sigmoid function as *f*_0_(*x*), *f*_1_(*x*), *f*_*i*_(*x*),..., *y*_0_, *y*_1_, and *y*_*i*_.
(4) The feedback errors for each layer are calculated separately.

$$\delta_{kl}^{\left(p\right)}=(d_{l}^{\left(p\right)}-y_{l}^{\left(p\right)})y_{l}^{\left(p\right)}(1-y_{l}^{\left(p\right)})$$ (2)

Here, **l* *= 0, 1, 2, …, *m*–1.


$$\begin{array}{c} \delta_{jk}^{\left(p\right)}=\sum_{l=0}^{m-1}\delta_{kl}^{\left(p\right)}\omega^{\prime }kl\prime {x_{k}^{\prime \prime }}^{\left(p\right)}(1-{x_{k}^{\prime \prime }}^{\left(p\right)}) \end{array}$$ (3)

Here, **k* *= 0, 1, 2, …, *n*_2_.


$$\begin{array}{c} \delta_{ij}^{\left(p\right)}=\sum_{k=0}^{n_{2}}\delta_{jk}^{\left(p\right)}\omega jk\prime {x_{j}^{\prime }}^{\left(p\right)}(1-{x_{j}^{\prime }}^{\left(p\right)}) \end{array}$$ (4)

Here, *j* = 0, 1, 2, …, *n*_1_.

The values of $\begin{array}{c} x_{k}^{\left(p\right)},\;x_{j}^{\left(p\right)} \end{array}$, and $x_{i}^{\left(p\right)}$ are recorded when transmitting the feedback signal.

(5) Determine whether the current number of training samples satisfies the application requirements. When *p* < *P*, iterative learning continues and the algorithm proceeds to step (2). If *p* = *P*, then iterative learning ends and the algorithm jumps to step (6).
(6) The connection weights of the network nodes are recalculated using the weight correction formula.
(7) Calculate *x*_*j*_’, *x*_*k*_’‘, and *y*_*l*_ according to the corrected weights. If, *p* and *l* reach $\left|d_{l}^{\left(p\right)}-y_{l}^{\left(p\right)}\right|<\varepsilon$ (ε is a minimum value) or the maximum training time is reached, then the algorithm ends. Otherwise, return to step (2) and continue iterative calculation.

### 4.2. Improvement of the BP network

The error function of the BP network has a significant impact on network performance. When the traditional network structure is fixed, the size and properties of the training dataset can lead to overfitting in the network. The weight attenuation method is the most commonly used error function optimisation approach. The core concept is to balance the output error (accuracy) and internal regularity (diversity) by adjusting the network weights, thereby enhancing the efficiency and quality of problem solving and suppressing overfitting. This paper establishes a regularisation method to mitigate overfitting and improve the error function of the traditional BP network [30–33]. The specific steps of the improved algorithm are as follows.

(1) Network initialisation: Initialise and assign values to parameters such as weights, biases, learning rates, balance coefficient, and maximum number of iterations.
(2) Input P learning samples in sequence. Let the current input be the Pth sample.
(3) Calculate the predicted values *f*_0_(*x*), *f*_1_(*x*), *f*_*i*_(*x*),..., *y*_0_, *y*_1_, and *y*_*i*_: Use the nonlinear activation Sigmoid function to calculate the output values of each layer.

$$g(x)=\frac{\text{1}}{1+\exp(x)}$$ (5)

(4)Optimise the overall error of the network: Accounting for both the ‘output accuracy’ and ‘internal diversity’‘ of the network, the overall error is evaluated using the following improved error function, thereby enhancing the efficiency and quality of problem solving.


Re\nolimitsg=γmse+(1−γ)msw (6)

Here, *γ* ∈ [0,1] is a balance coefficient and *mse* represents the mean squared error between network layers, which is an ‘output accuracy’ indicator used to measure the degree of deviation between the predicted values and actual values between network layers.

Between the input layer and the hidden layers, we use


$$mse_{1}=\frac{\text{1}}{P}\sum_{n=1}^{P}\frac{{\left(d_{1}^{\left(n\right)}-y_{1}^{\left(n\right)}\right)}^{2}}{\text{2}}.$$ (7)

The hidden layer extends to the output layer as


$$\begin{array}{c} mse_{l}=\frac{\text{1}}{P}\sum_{n=1}^{P}\frac{(d_{i}^{\left(n\right)}-y_{i}^{\left(n\right)}{)}^{2}}{2}. \end{array}$$ (8)

Here, P represents the sample size, n represents the sample number, *l* represents the sequence number of the hidden layer, d represents the actual value, and y represents the predicted value. This formula sums the mean squared differences between the actual and predicted values of each sample and then divides the result by the number of samples P. It reflects the average squared deviation between the predicted results of the network and the true results. The smaller the error, the better the prediction effect of the network.

The indicator *msw* represents the diversity of solutions or degree of dispersion of solutions. The larger the *msw* value, the wider the distribution of the solutions found by the algorithm. The smaller the *msw* value, the more concentrated the solution distribution. Different *msw* values can help the algorithm balance the diversity and convergence of searching, thereby improving the efficiency and quality of problem solving.


$$\begin{array}{c} msw=\frac{\text{1}}{N_{\omega }}\sum_{i=1}^{N_{\omega }}{\left(\omega_{i}\right)}^{2} \end{array}$$ (9)

Here, *N*_ω_ represents the number of adjustable neuron weights in the BP network and ω_*i*_ represents the neuron weight of the current network layer.

(5) Determine whether the current number of learning samples satisfies the application requirements. When *p* < *P*, iterative learning continues and the algorithm return to step (2). If *p* = *P*, then iterative learning ends and the algorithm proceeds to step (6).
(6) Calculate the gradient: Find the gradient of *Reg* with respect to the weight ω and update the parameters using gradient descent.

Total gradient of the hidden layer is


$$\begin{array}{c} \frac{\partial Re\mathrm{\backslash nolimits}g}{\partial \omega^{\left(1\right)}}=\gamma \cdot (y-d)\cdot g^{\prime }(z^{\left(l\right)})\cdot \omega^{\left(l\right)}+(1-\gamma )\cdot \frac{2\omega^{\left(1\right)}}{N_{\omega }}. \end{array}$$ (10)

Here, ***ω***^**(1)**^ is the weight parameter of the hidden layer, *g*′(*z*^*(l)*^) is the derivative of the activation function g(x) of the output layer; which is used to transfer the error gradient; *z*^*(l)*^ is the weighted sum of the output layers of the network; ***a***^(1)^ represents the output activation value of the hidden layer; and $\frac{2\omega^{\left(1\right)}}{N_{\omega }}$ represents the gradient of the L2 regularisation term with respect to the hidden layer weights ***ω***^**(1)**^, penalising larger weights to force them to shrink.

Total gradient of the output layer is


$$\frac{\partial Re\mathrm{\backslash nolimits}g}{\partial \omega^{\left(l\right)}}=\gamma \cdot (y-d)\cdot g\prime (z^{\left(l\right)})\cdot \alpha^{\left(1\right)}+(1-\gamma )\cdot \frac{2\omega^{\left(l\right)}}{N_{\omega }}.$$ (11)

Here, ***ω***^***(l)***^ is the weight parameter of the output layer and $\frac{2\omega^{\left(l\right)}}{N_{\omega }}$ represents the gradient of the L2 regularisation term with respect to the output layer weights ***ω***^***(l)***^.

(7) Update the weight and balance coefficient: Update ω and γ in accordance with the principle of gradient descent.

$$\omega_{new}=\omega_{old}-\eta \cdot \frac{\partial Re\mathrm{\backslash nolimits}g}{\partial \omega }$$ (12)

$$\gamma_{new}=\gamma_{old}-\eta \cdot \frac{\partial Re\mathrm{\backslash nolimits}g}{\partial \gamma }$$ (13)

Here, ωnew, γnew respectively represent the weights and balance coefficients of the updated network parameters; ωold, γold respectively represent the weights and balance coefficients of the previous network parameters; and η represents the learning rate, which controls the ‘step size’ of the parameter updates. It determines how strongly the parameters should be updated after calculating the parameter gradient.

(8) Calculate xj’, xk’‘, and y*l* according to the corrected weights. If, p and *l* reach $\left|d_{l}^{\left(p\right)}-y_{l}^{\left(p\right)}\right|<\varepsilon$ (ε is a minimum value) or the maximum number of iterations is reached, then the algorithm ends. Otherwise, return to step (2) and continue iterative calculation.

**Fig 15:**
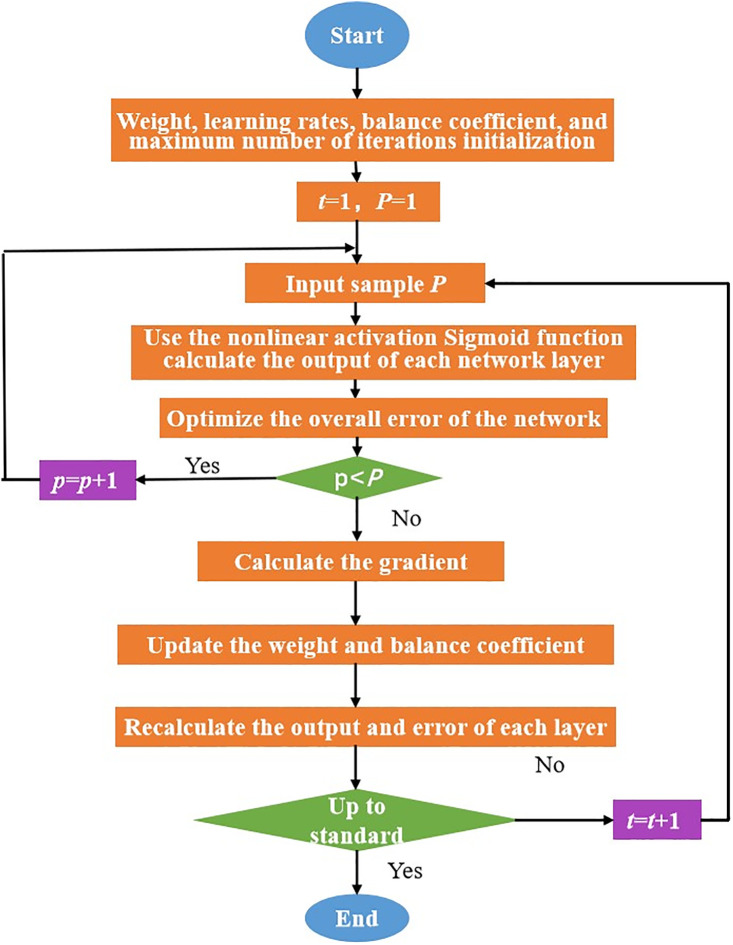
Improved BP network algorithm flow.

The improved algorithm flow is illustrated in Fig 15.

## 5. BP neural network model and engineering prediction of the axial compression properties of STCDSRAC

### 5.1. BP neural network model for the axial compressive properties of STCDSRAC

**Fig 16:**
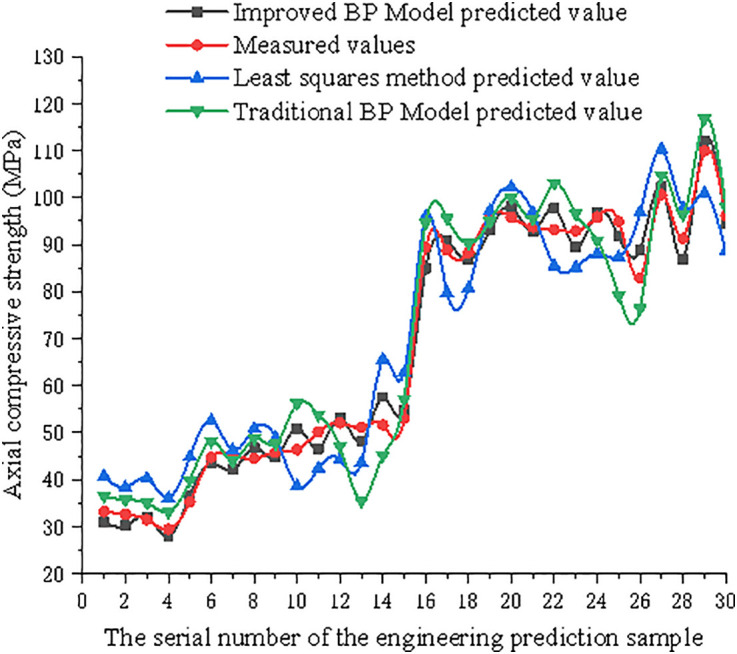
Comparison of improved model prediction values, traditional model prediction values, least squares method prediction values, and measured values of axial compressive strength.

**Fig 17:**
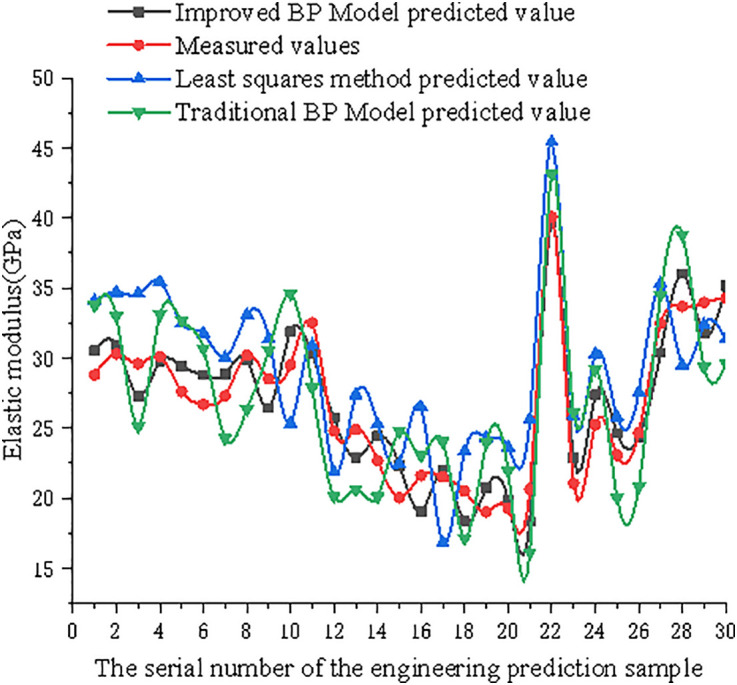
Comparison of improved model prediction values, traditional model prediction values, least squares method prediction values, and measured values of the elastic modulus.

To predict the axial compression properties of STCDSRAC to guide engineering practice [34,35], 161 sets of sample data of concrete specimens were selected as a modelling dataset, with or without steel constraints and with different desert sand contents and water–cement ratios. The 161 sets of data were divided into a training group, validation group, and prediction group. Specifically, 113 samples were used as a training set to update the model parameters, 18 samples were used for verification to avoid overfitting, and 30 samples were used for final performance evaluation. Stratified sampling was adopted for data division and a fixed random seed was used to ensure the reproducibility of the division results. To eliminate the random influence of data partitioning, K-fold cross-validation was applied. Considering the size of the sample space, two intermediate hidden layers were used in the BP neural network with 64 and 32 neurons. After training, the improved BP neural network model was used to predict the axial compressive strength and elastic modulus of concrete specimens. The training verification results are presented in Tables 5 and 6, and the corresponding BP prediction results are presented in Figs 16 and 17. The network learning rate was 0.15, initial balance coefficient was 0.2, maximum number of iterations was 500, ε was 0.001, and training error threshold was < 10%. To preserve the generality of the model input parameters, the steel constraint parameter value was set to zero for no steel constraint and set to the wall thickness of the tube for steel constraint.

**Table 5:** Training verification results for the axial compressive strength of STCDSRAC.

Specimen number	Input parameters			Training verification values and errors of axial compressive strength		
	Steel constraint	Desert-sand content (%)	Water–cement ratio (%)	Training verification value (MPa)	Measured value (MPa)	Error (%)
1	0	20	30	51.08	53.40	4.54
2	0	20	35	49.77	52.19	4.86
3	0	20	40	45.25	44.61	−1.41
4	0	40	30	47.53	49.12	3.35
5	0	40	35	46.78	45.29	−3.19
6	0	40	40	43.55	44.85	2.99
7	0	60	30	44.61	47.75	7.03
8	0	60	35	45.89	43.70	−4.77
9	0	60	40	43.77	44.77	2.28
10	3	20	30	93.7	89.16	−4.84
11	3	20	35	92.3	95.82	3.81
12	3	20	40	90.5	89.42	−1.19
13	3	40	30	94.64	97.78	3.32
14	3	40	35	95.5	89.52	−6.26
15	3	40	40	93.88	92.09	−1.91
16	3	60	30	87.42	91.85	5.07
17	3	60	35	87.67	85.52	−2.45
18	3	60	40	86.98	88.57	1.83

**Table 6:** Training verification results for the elastic modulus of STCDSRAC.

Specimen number	Input parameters			Training verification values and errors of elastic modulus		
	Steel constraint	Desert-sand content (%)	Water–cement ratio (%)	Training verification Value (GPa)	Measured value (GPa)	Error (%)
1	0	20	30	29.37	27.65	−5.86
2	0	20	35	27.58	26.94	−2.31
3	0	20	40	25.46	27.77	9.09
4	0	40	30	30.77	31.14	1.19
5	0	40	35	29.76	27.94	−6.10
6	0	40	40	26.11	24.01	−8.04
7	0	60	30	30.88	29.32	−5.06
8	0	60	35	31.67	32.01	1.06
9	0	60	40	28.25	30.32	7.33
10	3	20	30	34.2	31.85	−6.88
11	3	20	35	32.78	35.01	6.79
12	3	20	40	30.88	29.95	−3.02
13	3	40	30	34.3	32.34	−5.71
14	3	40	35	33.21	31.46	−5.26
15	3	40	40	31.05	28.70	−7.58
16	3	60	30	36.3	38.89	7.14
17	3	60	35	37.1	36.65	−1.22
18	3	60	40	34.2	31.33	−8.40

### 5.2. Training of the improved BP neural network model

### 5.3. Prediction accuracy

As shown in Tables 5 and 6, and Figs 16 and 17, compared with the fitted values of the least squares method and predicted values of the traditional BP neural network model, the improved BP neural network prediction values are closer to the measured values of the axial compressive strength and elastic modulus. After training with relevant sample data, the improved BP model accurately characterised the uncertainty distribution relationship between the inputs and outputs, indicating that it can be used to predict the axial compressive strength and elastic modulus of STCDSRAC in practical applications.

### 5.4. Comparison of benchmark test

To demonstrate the superiority of the improved BP network, it was tested and compared with popular benchmark methods in the field of small data regression (ridge/LASSO, SVR, RF, XGBoost), as well as the standard L2-regularised BP neural network. To ensure data consistency, all tests used the same sample dataset. We adopted unified evaluation indicators and settings such as the same training division and cross-validation strategies to ensure the fairness and reproducibility. The termination conditions for the tests were ‘500 iterations’ or ‘error less than 0.001’. The benchmark methods for small data regression and relevant parameters for the standard L2-regularised BP neural network are listed in Table 7.

**Table 7:** Relevant parameters of the benchmark models.

Benchmark model	Parameter setting
BP with standard L2 regularisation	Regularisation coefficient: λ∈[0.001,0.01,0.1]; 10 fold cross-validation; The learning rate and number of iterations are consistent with the improved BP network (control variable).
Ridge	Regularisation intensity: α∈[0.01,0.1,1,10]; Grid search + 10 fold cross-validation
LASSO	Regularisation intensity: α∈[0.001,0.01,0.1,1]; Grid search + 10 fold cross-validation
SVR	Kernel function: RBF (Commonly used for nonlinear fitting of small data); Penalty coefficient: C∈ [1,5,10]; Kernel parameters: γ∈[0.1,1,10]
RF	Number of decision trees: 100; Maximum depth: 10 fold cross-validation control complexity
XGBoost	Learning rate: 0.1; Number of trees: 100; Regularisation coefficient: λ = 1 (cross-validation optimisation)

**Fig 18:**
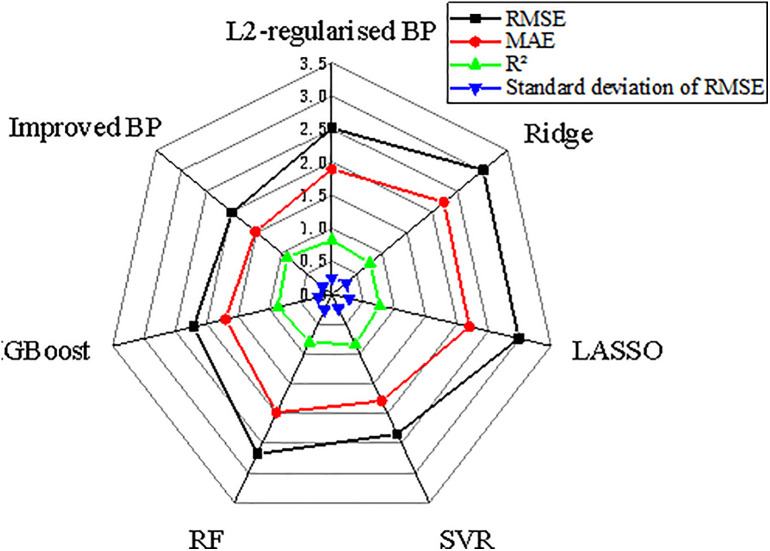
Performance comparison between the improved BP model and benchmark models for small data regression.

We considered root-mean-squared error (RMSE, reflecting the overall error), mean absolute error (MAE, resistant to outlier interference), R² (the closer to one, the better the fitting effect), and the standard deviation of RMSE in K-fold cross-validation (the smaller the value, the more stable the model) as performance indicators. A performance comparison of each model on the small data regression task is presented in Fig 18. One can see that compared with the benchmark methods for small data regression and classic L2-regularised BP neural network, the improved BP model has comprehensive advantages in terms fitting accuracy, overfitting mitigation, and stability.

### 5.5. Algorithm efficiency

**Fig 19:**
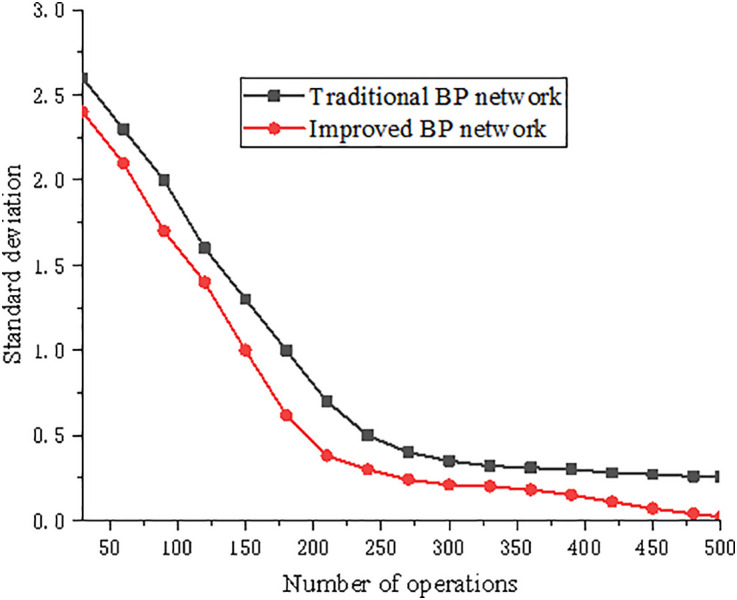
Characteristic chart of algorithm efficiency.

The improved BP neural network algorithm and traditional BP algorithm were used to predict the axial compressive strengths and elastic moduli of concrete specimens and their efficiencies were compared based on 500 iterative operations. The results are presented in Fig 19. The configuration of the experimental platform was as follows: host CPU, Intel Core I5-6500; main frequency, 3.2 GHz; memory, DDR256G; hard disk, 1 TB; network card, 1000 M; operating system, Windows XP SP3; debugging software, MATLAB 2010.

With an increase in the number of iterations, the improved BP neural network algorithm converged more rapidly with smaller errors. Therefore, it can be concluded that compared with the traditional algorithm, the proposed algorithm is more robust and efficient.

## 6. Conclusion

(1) Compared with the corresponding DSRAC samples, the peak stress and peak strain of the STCDSRAC samples were significantly improved, indicating that steel tube confinement can significantly improve the ductility of DSRAC and enhance its deformation performance. As a result of the small particle size and smooth surface of desert sand, its filling effect in concrete is poor. Therefore, as the desert sand content increases from 20% to 60% and water–cement ratio increases from 30% to 40%, the peak stress of STCDSRAC decreases slightly. Additionally, it was found that when desert sand is used in conjunction with recycled aggregates, it will introduce significant engineering uncertainties into the mechanical properties of concrete.
(2) The axial compressive strength and elastic modulus of STCDSRAC exhibited little change during the elastic stage under reciprocating loading. In the initial stage of loading, the lateral strain exhibited discrete characteristics, mainly because the deformation of the specimens was small, and the engineering error had a significant influence on the results. However, in the stage close to the peak stress, as a result of the numerous micro-cracks that accumulated inside the specimen, the transverse deformation increased rapidly, increasing the transverse coefficient. As the reciprocating loading specimen entered the elastic–plastic stage, its unloading stiffness decreased.
(3) The axial compressive strength and elastic modulus exhibited engineering uncertainty as a result of the comprehensive influence of the steel constraint, desert sand content, and water–cement ratio. Based on the evaluation function of the balance coefficients and interlayer mean squared error, an improved BP neural network model for the axial compressive properties of STCDSRAC was established. The model considers the presence or absence of steel constraints, desert sand content, and the water–cement ratio as inputs and provides the axial compression strength and elastic modulus as outputs. The proposed model provides a new method for the uncertainty analysis of the engineering mechanical properties of STCDSRAC.
(4) The improved BP neural network model can predict the axial compressive properties of STCDSRAC under actual operating conditions. Compared with the predicted values of the least squares method and traditional BP neural network model, the predicted values of the improved BP neural network were closer to the measured values. The proposed model provides comprehensive advantages in terms fitting accuracy, overfitting mitigation, and stability.

## Supporting information

S1 Dataset Training data of the axial compressive strength model of desert sand recycled aggregate concrete under steel pipe constraints. (XLSX)

S2 Dataset Model training data of elastic modulus of desert sand recycled aggregate concrete under steel pipe constraints. (XLSX)

## References

[1] Josef K. Evaluation of the combination of desert sand and calcium sulfoaluminate cement for the production of concrete. Constr Build Mater. 2020;243:118281.

[2] Yan WL, Wu G, Dong Z h q. Optimization of the mix proportion for desert sand concrete based on a statistical model. Constr Build Mater. 2019;226:469–82.

[3] Ji Y, Qasem MGS, Xu T, Mohammed AOY. Mechanical properties investigation on recycled rubber desert sand concrete. J CO2 Utili. 2024;88:102939. doi: 10.1016/j.jcou.2024.102939

[4] Wafa H, Dalila B, Ammar N. Statistical modeling of physical and mechanical responses of roller-compacted sand concrete made with ternary sand using the experimental design method. Constr Build Mater. 2022;345:128354.

[5] Shi Z. Green manufacturing of silicate materials using desert sand as a raw-material resource. Construction and Building Materials. 2022;338:127539. doi: 10.1016/j.conbuildmat.2022.127539

[6] Akhtar MN, Jameel M, Ibrahim Z, Muhamad Bunnori N, Bani-Hani KA. Development of sustainable modified sand concrete: An experimental study. Ain Shams Engineering Journal. 2024;15(1):102331. doi: 10.1016/j.asej.2023.102331

[7] Hou L, Zhang X, Huang W, Zhang X. Uniaxial Compression Behavior of Polypropylene and Glass Fiber-Reinforced Desert Sand Concrete. Arab J Sci Eng. 2024. doi: 10.1007/s13369-024-09875-w

[8] Qu C, Qin Y, Luo L, Zhang L. Mechanical properties and acoustic emission analysis of desert sand concrete reinforced with steel fiber. Sci Rep. 2022;12(1):20488. doi: 10.1038/s41598-022-24198-2 36443411 PMC9705708

[9] Zhang X, Zhu Y, Zhang W. Research on axial compression performance of recycled aggregate concrete-filled steel tubular column. J Constr S Res. 2025;229:109541.

[10] Yuan H, Chen Y, Kang L, Geng T, Ma K. Compressive axial load performance of GFRP–confined recycled aggregate concrete–filled steel tube stub columns. Journal of Constructional Steel Research. 2024;220:108835. doi: 10.1016/j.jcsr.2024.108835

[11] Wang J, Li H. Compression Performance of Steel Tube with Recycled Large Aggregate Self-compacting Concrete. Iran J Sci Technol Trans Civ Eng. 2023;48(4):2003–18. doi: 10.1007/s40996-023-01281-w

[12] Fábio C, Isabel V, João A, Ana N. Experimental characterization of bond between concrete and HDG steel tubes for mixed steel-concrete structures. Mater Struct. 2024;57:89.

[13] Liu J, Du J, Gao S, Wang H. Multiscale Study on the Axial Compression Performance of PET FRP–Concrete–Steel Double-Skin Tubular Stub Columns. Int J Civ Eng. 2024;22(9):1659–78. doi: 10.1007/s40999-024-00954-5

[14] Hao X-K, Zheng J-J, Fu C, Wang Y, Feng Q. Passive stress-strain model and ultimate strength prediction for short steel tube confined concrete (STCC) columns allowing for size effect. Case Studies in Construction Materials. 2024;20:e02866. doi: 10.1016/j.cscm.2024.e02866

[15] Chen R-S, Zhang H-Y, Hao X-K, Yu H-X, Shi T, Zhou H-S, et al. Experimental study on ultimate bearing capacity of short thin-walled steel tubes reinforced with high-ductility concrete. Structures. 2024;68:107109. doi: 10.1016/j.istruc.2024.107109

[16] Gong L, Zhao X, Bu Y, Xu T, Yu XB, Liang YT. Research on frost resistance of desert sand machine-made sand blended concrete and life prediction. Structures. 2024;70:107875.

[17] Zhang M, Hu P, Sun S, Yao J, Zhong J. Ultra-high-performance alkali-activated concrete produced with desert sand incorporations. Constr Build Mater. 2025;476:141240.

[18] Xu W, Liu H, Qin D, Doh SI. Study on the mechanical properties of desert sand concrete under dry-wet cycles with sulfate erosion. Physics and Chemistry of the Earth, Parts A/B/C. 2025;138:103852. doi: 10.1016/j.pce.2025.103852

[19] Shen Y, Peng C, Hao J, Bai Z, Li Y, Yang B. High temperature resistance of desert sand concrete: Strength change and intrinsic mechanism. Constr Build Mater. 2022;327:126948.

[20] Zeng Z, Ren G. Experimental study on axial compression performance of steel tube confined concrete after freeze-thaw cycles. Results in Engineering. 2024;24:103267. doi: 10.1016/j.rineng.2024.103267

[21] Hui C, Wang Y, Ma Z, Lei S, Hai R. Investigation on axial compression performance of self-compacting fly ash concrete filled square steel tube column: Tests and numerical simulation. Case Studies in Construction Materials. 2024;20:e03281. doi: 10.1016/j.cscm.2024.e03281

[22] Hasan HG, Ekmekyapar T, Shehab BA. Mechanical performances of stiffened and reinforced concrete-filled steel tubes under axial compression. Marine Structures. 2019;65:417–32. doi: 10.1016/j.marstruc.2018.12.008

[23] Alrebeh SK, Ekmekyapar T. Structural performance of short concrete-filled steel tube columns with external and internal stiffening under axial compression. Structures. 2019;20:702–16. doi: 10.1016/j.istruc.2019.06.015

[24] Chen X, Zhao D, Yan Y, Lu Y, Li S. Experimental study and parameter sensitivity analysis of axial compression in locally corroded slender circular steel tubes. Constr Build Mater. 2025;474:141077.

[25] Zhang Z, Guo B, Zhu C. Experimental study on shear mechanical characteristics and its size effect of concrete joints based on BP neural network method. Constr Build Mater. 2024;442:137583.

[26] AL-Bukhaiti K, Liu Y, Zhao S, Abas H. An Application of BP Neural Network to the Prediction of Compressive Strength in Circular Concrete Columns Confined with CFRP. KSCE Journal of Civil Engineering. 2023;27(7):3006–18. doi: 10.1007/s12205-023-1542-6

[27] Zhong W, Ding H, Zhao X, Fan L. Mechanical properties prediction of geopolymer concrete subjected to high temperature by BP neural network. Constr Build Mater. 2023;409:133780.

[28] Li Q, Su R, Qiao H, Su L, Wang P, Gong L. Prediction of compressive strength and porosity of vegetated concrete based on hybrid BP neural networks. Materials Today Communications. 2025;44:112080. doi: 10.1016/j.mtcomm.2025.112080

[29] Haykin S. Neural Networks and Learning Machines. 3rd ed. London: Pearson Press; 2008.

[30] Yan J. Application of improved BP neural network model in bank financial accounting. Intelligent Systems with Applications. 2022;16:200155. doi: 10.1016/j.iswa.2022.200155

[31] Wang XG, Tang Z, Tamura H, Ishii M. A modified error function for the backpropagation algorithm. Neurocomputing. 2004;57:477–84. doi: 10.1016/j.neucom.2003.12.006

[32] Lee H-M, Chen C-M, Huang T-C. Learning efficiency improvement of back-propagation algorithm by error saturation prevention method. Neurocomputing. 2001;41(1–4):125–43. doi: 10.1016/s0925-2312(00)00352-0

[33] Rumelhart DE, Hinton GE, Williams RJ. Learning representations by back-propagating errors. Nature. 1986;323(6088):533–6. doi: 10.1038/323533a0

[34] Zheng N-H, Zhang W-P, Zhou Y, Liu Y. Confinement strength prediction of corroded rectangular concrete columns using BP neural networks and support vector regression. Structures. 2024;67:107021. doi: 10.1016/j.istruc.2024.107021

[35] Feng Q, Xie X, Wang P, Qiao H, Zhang Y, Ma Y. Prediction of durability of reinforced concrete based on hybrid-Bp neural network. Constr Build Mater. 2024;425:136091.

